# Dissection of broad-spectrum resistance of the Thai rice variety Jao Hom Nin conferred by two resistance genes against rice blast

**DOI:** 10.1186/s12284-017-0159-0

**Published:** 2017-05-11

**Authors:** Chaivarakun Chaipanya, Mary Jeanie Telebanco-Yanoria, Berlaine Quime, Apinya Longya, Siripar Korinsak, Siriporn Korinsak, Theerayut Toojinda, Apichart Vanavichit, Chatchawan Jantasuriyarat, Bo Zhou

**Affiliations:** 1 Department of Genetics, Faculty of Science, Kasetsart University, Chatuchak, Bangkok 10900 Thailand; 2 Genetics and Biotechnology Division, International Rice Research Institute, Los Baños, Laguna 4031 Philippines; 3 Rice Gene Discovery Laboratory, National Center for Genetic Engineering and Biotechnology (BIOTEC), National Science and Technology Development Agency, Pathum Thani, 12120 Thailand; 4 Plant Biotechnology Research Unit, National Center for Genetic Engineering and Biotechnology (BIOTEC), National Science and Technology Development Agency, Pathum Thani, 12120 Thailand; 5 Rice Science Center, Kasetsart University, Kamphaeng Saen, Nakhon Pathom, 73140 Thailand; 6 Agronomy Department Faculty of Agriculture at Kamphaeng Saen, Kasetsart University, Nakhon Pathom, 73140 Thailand; 7 Center for Advanced Studies in Tropical Natural Resources, National Research University-Kasetsart University (CASNAR, NRU-KU), Chatuchak, Bangkok 10900 Thailand

**Keywords:** *Magnaporthe oryzae*, Quantitative trait loci (QTL), Durable resistance

## Abstract

**Background:**

Rice (*Oryza sativa*) is one of the most important food crops in the world. Rice blast, caused by the fungal pathogen *Magnaporthe oryzae*, is one of the most destructive rice diseases worldwide. To effectively cope with this problem, the use of rice blast resistance varieties through innovative breeding programs is the best strategy to date. The Thai rice variety Jao Hom Nin (JHN) showed broad-spectrum resistance against Thai rice blast isolates. Two QTLs for blast resistance in JHN were reported on chromosome 1 (*QTL1*) and 11 (*QTL11*).

**Results:**

Monogenic lines of *QTL1* (QTL1-C) and *QTL11* (QTL11-C) in the CO39 genetic background were generated. Cluster analysis based on the disease reaction pattern of QTL1-C and QTL11-C, together with IRBLs, showed that those two monogenic lines were clustered with IRBLsh-S (*Pish*) and IRBL7-M (*Pi7*), respectively. Moreover, sequence analysis revealed that *Pish* and *Pi7* were embedded within the *QTL1* and *QTL11* delimited genomic intervals, respectively. This study thus concluded that *QTL1* and *QTL11* could encode alleles of *Pish* and *Pi7*, designated as *Pish-J* and *Pi7-J*, respectively. To validate this hypothesis, the genomic regions of *Pish-J* and *Pi7-J* were cloned and sequenced. Protein sequence comparison revealed that Pish-J and Pi7-J were identical to Pish and Pi7, respectively. The holistic disease spectrum of JHN was found to be exactly attributed to the additive ones of both QTL1-C and QTL11-C.

**Conclusion:**

JHN showed broad spectrum resistance against Thai and Philippine rice blast isolates. As this study demonstrated, the combination of two resistance genes, *Pish-J* and *Pi7-J*, in JHN, with each controlling broad-spectrum resistance to rice blast disease, explains the high level of resistance. Thus, the combination of *Pish* and *Pi7* can provide a practical scheme for breeding durable resistance in rice against rice blast disease.

**Electronic supplementary material:**

The online version of this article (doi:10.1186/s12284-017-0159-0) contains supplementary material, which is available to authorized users.

## Background

Rice is one of the most important staple food crops in the world. The increasing world population leads to increased demand for rice production. In order to feed the growing population, rice varieties that are resistant to known diseases and that can produce high yields should be used (Khush and Jena 2009). Rice blast, caused by an ascomycete *Magnaporthe oryzae*, is one of the most destructive diseases of rice worldwide (Ahn et al. 2000), which leads to economic losses of more than 70 billion dollars (Scheuermann et al. 2012). In many Asian countries, the outbreak of rice blast which led to complete losses in rice production has been reported (Sobrizal and Anggiani 2007; Variar 2007; Lei et al. 2016; Hairmansis et al. 2016; Khan et al. 2016; Nguyet et al. 2016).

The most powerful tool to control rice blast is to use resistant varieties. These varieties protect rice plants against the pathogens, upon infection, via the induction of hypersensitive response (HR), which occurs via gene-for-gene recognition of a pathogen effector (Avr) and a host-encoded resistance (R) protein (Gururani et al. 2012). The majority of plant *R* genes encode proteins that have putative central nucleotide-binding-site (NBS) and carboxy-terminal leucine-rich repeats (LRRs). These NBS-LRR proteins are divided into two major classes: the first class has an N-terminal domain which shares homology with the mammalian toll-interleukin-1-receptor (TIR) domain while the second class encodes an amino-terminal coiled-coil motif (CC-NBS-LRR) (DeYoung and Innes 2006; Meyers et al. 2003). To date, there are four types of coding proteins of *R* genes against rice blast: (1) NBS-LRR protein; (2) CC-NBS-LRR protein; (3) B-lectin binding and intracellular serine–threonine kinase domain protein; and (4) proline-rich protein (Chen et al. 2006; Zhou et al. 2006; Ashikawa et al. 2008; Fukuoka et al. 2009). The first rice blast resistance gene was cloned by Wang et al. 1999 and more than 20 *R* genes were cloned since then. These blast resistance genes are not randomly positioned on rice chromosomes (Sharma et al. 2012). Many *R* genes were cloned from chromosomes 6, 11, and 12. Some are clustered on a gene family (Sharma et al. 2012). Many allelic genes have been reported, for example *Pish/Pi35* on chromosome 1 (Takahashi et al. 2010; Fukuoka et al. 2014), *Pi9/Pi2/Pizt/Pi50* on chromosome 6 (Qu et al. 2006; Zhou et al. 2006; Su et al. 2015), *Pi5/Pii* on chromosome 9 (Lee et al. 2009; Takagi et al. 2013), and *Pikh/Pikm/Pik/Pikp/Pi1* on chromosome 11 (Ashikawa et al. 2008; Xu et al. 2008; Yuan et al. 2011; Zhai et al. 2011; Hua et al. 2012). To date, 11 avirulence (*Avr*) genes in rice blast fungus have been cloned. Nine out of 11 genes, namely; *AvrPita* (Orbach et al. 2000), *ACE1* (Bohnert et al. 2004), *AvrPia*, *AvrPii*, *AvrPik* (Yoshida et al. 2009), *AvrPiz-t* (Li et al. 2009), *Avr1-CO3*9 (Zheng et al. 2011), *AvrPib* (Zhang et al. 2015), and *AvrPi9* (Wu et al. 2015), have their corresponding *R* genes in rice. However, there are no known *R* genes in rice for *PWL1* (Kang et al. 1995) and *PWL2* (Sweigard et al. 1995). There are many scenarios that describe the specificity of recognition between resistance proteins and avirulence proteins. For example, one *R* gene can recognize more than one *Avr* gene, an example of which is *Pia* that can recognize both *AvrPia* and *Avr1-CO39* (Cesari et al. 2013), or it could also be that many *R* genes can recognize the same *Avr* gene, as in the case of *AvrPik*-D that can be recognized by *Pik, Pikm*, and *Pikp* (Yoshida et al. 2009). In another case, the presence of two *R* genes is required for the mediated resistance to rice blast: *Pi-km1* and *Pi-km2* from Tsuyuake (Ashikawa et al. 2008) and *Pi5-1* and *Pi5-2* from RIL260 (Lee et al. 2009).

Genes conferring broad-spectrum resistance are necessary in breeding programs. The presence of even only one *R* gene can induce resistance to many isolates of *M. oryzae* (Qu et al. 2006). Many broad-spectrum *R* genes have been validated such as *Pi9* (Qu et al. 2006), *Pi2*, *Piz-t* (Zhou et al. 2006), *Pi40* ^*(t)*^ (Jeung et al. 2007), *Pi20* ^*(t)*^ (Li et al. 2008), *Pi5* (Lee et al. 2009), *Pi1* (Hua et al. 2012), *Pi54rh* (Das et al. 2012), *Pi56* ^*(t)*^ (Liu et al. 2013), and *Pi50* (Su et al. 2015). Unfortunately, the resistance controlled by most race-specific *R* genes is prone to collapse often in a few years after introduction to the rice field due to the quick adaptation of rice blast pathogen (Kiyosawa 1982; Fukuoka et al. 2009).

Many strategies to keep the durability of resistance have been suggested, including multi-varietal planting, pyramiding of major *R* genes, and using partial resistance genes (Khush and Jena 2009). The use of partial resistance genes is one way to maintain the resistance of *R* gene(s) in the field. Partial resistance genes such as *Pi34* (Zenbayashi-Sawata et al. 2005), *pi21* (Fukuoka et al. 2009), and *Pi35* (Fukuoka et al. 2014) have been reported. The pyramiding of partial resistance genes for enhancing the durability of resistance has been reportedly used in several breeding programs (Yasuda et al. 2015).

Jao Hom Nin (JHN) is a Thai rice variety which shows broad-spectrum resistance against rice blast in Thailand. Only two out of 346 blast isolates collected from all the rice growing areas in Thailand can overcome the resistance of JHN (Sreewongchai 2008). Moreover, seven out of 124 blast isolates collected worldwide can overcome the resistance of JHN, two of the isolates are from Colombia while five are from China (Sreewongchai 2008). Two Quantitative Trait Loci (QTLs) which are associated with rice blast resistance were mapped on chromosomes 1 and 11 of JHN (Noenplab et al. 2006). The major QTL (*QTL11*) that controls complete resistance was mapped on chromosome 11 by the SSR markers RM144 and RM224. The minor QTL (*QTL1*) which confers partial resistance was mapped on chromosome 1 by the flanking markers RM319 and RM 212 (Noenplab et al. 2006). *QTL11* is located on the *Pik* locus which is a gene family locus. Interestingly, *QTL1* is located on the *Pish* locus which is composed of *Pi37* (Lin et al. 2007), *Pish* (Takahashi et al. 2010), *Pi35* (Fukuoka et al. 2014), and *Pi64* (Ma et al. 2015). In Thailand, JHN is used as a rice blast resistant donor in breeding programs. Introgressions of *QTL1* and *QTL11* from JHN via marker-assisted selection (MAS) into susceptible cultivars such as KDML105 (Noenplab et al. 2006) and RD6 (Wongsaprom et al. 2010) were successfully accomplished. In this study, the resistance gene/s in both *QTL1* and *QTL11* of JHN, which confer broad-spectrum resistance against rice blast populations from Thailand and the Philippines, were cloned and characterized and their disease spectrum was validated. Revealing the JHN rice blast resistance genes discloses the powerful resistance gene combination for resistance and durability to rice blast fungus populations in Thailand, the Philippines, and worldwide.

## Results

### JHN controls broad-spectrum resistance through the combination of *QTL1* and *QTL11*

**Fig. 1:**
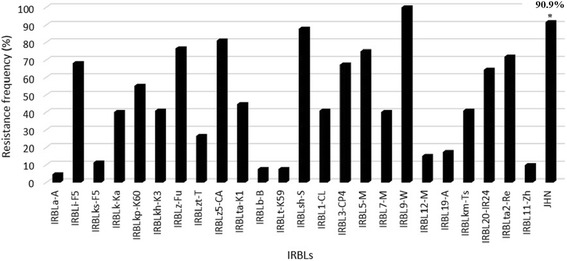
The resistance frequency of IRBLs and JHN against Philippine rice blast isolates. The resistance frequency of each rice variety was calculated based on the resistance reactions against 132 Philippine isolates as listed in Additional file 1: Table S1. The resistance frequency of JHN was indicated on the top of the bar

To evaluate the resistance spectrum of JHN, 132 diverse blast isolates collected from the Philippines were used for disease assessment. It was found that JHN showed resistance to 120 out of the 132 isolates, representing 90.9% resistance frequency (Fig. 1 & Additional file 1: Table S1). The reactions of different IRRI-bred blast resistant lines (IRBLs) to these isolates were also assessed, revealing that each line had a varied resistance frequency (Fig. 1). Compared to the majority of IRBLs, JHN controlled a quite high resistance frequency to the Philippine isolates. The data indicated that JHN was a promising rice line controlling broad-spectrum resistance to the Philippine isolates as to the Thai isolates (Sreewongchai 2008). Given the fact that JHN contained at least two resistance loci, i.e., *QTL1* on chromosome 1 and *QTL11* on chromosome 11 (Noenplab et al. 2006), we generated two monogenic lines which were designated as QTL1-C and QTL11-C containing individual *QTL1* and *QTL11*, respectively, in the CO39 genetic background via marker aided backcrossing (MABC) (Additional file 2: Figure S1). The derived homozygous BC_3_F_3_ monogenic lines were used for the spectrum analysis against 42 representative isolates selected from the set of 132 isolates as described above. Eight isolates showed virulence to QTL1-C, QTL11-C, and JHN, indicating that neither *QTL1* nor *QTL11* controlled resistance to these eight isolates (Table 1). Out of 34 avirulent isolates to JHN, three were virulent to QTL1-C but avirulent to QTL11-C, suggesting that *QTL11* rather than *QTL1* contributed to the resistance of JHN to these three isolates (Table 1). On the contrary, 12 isolates were avirulent to QTL1-C but virulent to QTL11-C, suggesting that *QTL1* rather than *QTL11* contributed to the resistance of JHN to these 12 isolates (Table 1). The remaining 19 isolates were avirulent to QTL1-C, QTL11-C, and JHN, suggesting that both *QTL1* and *QTL11* controlled the resistance to these 19 isolates (Table 1). We thus concluded that the resistance of JHN to these 34 isolates was additively controlled by *QTL1* and *QTL11*.

**Table 1:** The disease spectrum of QTL1-C, QTL11-C, IRBLsh-S, IRBL7-M, and JHN against 42 representative Philippine blast isolates

Isolates	Resistance reactions of different rice lines to a set of 42 representative isolates				
	QTL1-C	IRBLsh-S	QTL11-C	IRBL7-M	JHN
5008-3	R	R	R	R	R
5092-3	R	R	R	R	R
5167-1	R	R	S	S	R
9244-3	S	S	R	R	R
9406-3	R	R	R	R	R
9475-1	R	R	R	R	R
9482-1	R	R	S	S	R
9497-3	S	S	R	R	R
PO6-6	R	R	R	R	R
CA89	R	R	R	R	R
IK81-3	R	R	R	R	R
IK81-25	R	R	S	S	R
JMB8401	S	S	S	S	S
JMB840610	R	R	S	S	R
M101-1-2-9-1	R	R	R	R	R
M64-1-3-9-1	S	S	S	S	S
PO83-Z1-30	R	R	R	R	R
V86010	R	R	R	R	R
BN111	R	R	R	R	R
BN209	R	R	R	R	R
MO15-2	R	R	R	R	R
MO15-6	S	S	S	S	S
MO15-20	R	R	S	S	R
MO15-21	R	R	R	R	R
MO15-24	S	S	R	R	R
MO15-32	R	R	R	R	R
MO15-51	R	R	R	R	R
MO15-56	R	R	R	R	R
MO15-64	R	R	R	R	R
MO15-102	R	R	R	R	R
MO15-105	R	R	S	S	R
MO15-106	R	R	S	S	R
MO15-138	S	S	S	S	S
MO15-144	R	R	S	S	R
MO15-148	S	S	S	S	S
MO15-200	R	R	S	S	R
Pi9-G7-1 V-1	R	R	S	S	R
Pi9-G7-1I-1	R	R	S	S	R
Pi9-G7-1H-1	R	R	S	S	R
Pi9-G7-1 W-1	S	S	S	S	S
Pi9-G7-1B-1	S	S	S	S	S
Pi9-G7-1E-1	S	S	S	S	S

### Genetic validation of genomic location of *QTL1* and *QTL11* in JHN

**Fig. 2:**
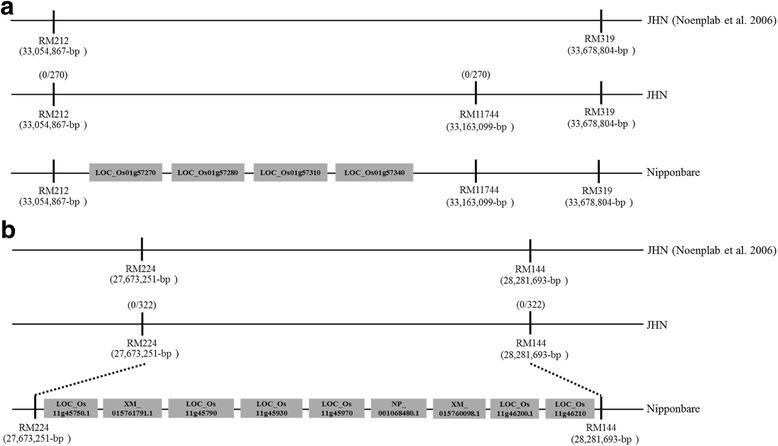
An integrated genetic and physical map of *QTL1* (**a**) and *QTL2* (**b**). The chromosomal position of SSR markers delimiting the genomic interval of QTLs by referring to the one in the reference genome of Nipponbare was indicated below the line. The number of recombinants/toal F_3_ progeny at each SSR marker was indicated above the line. Four and 9 NBS-LRR type R genes predicted in the genomic intervals in Nipponbare for QTL1 and QTL11, respectively, were indicated in boxes with gene IDs. The figure was not draw in scale

To validate the genetic delimitation of *QTL1* and *QTL11* in JHN, two F_3_ populations were generated as illustrated in (Additional file 2: Figure S1), each derived from a single heterozygous F_2_ plant containing an individual QTLs determined by the flanking simple sequence repeat (SSR) markers of *QTL1* (RM212 and RM11744) and of *QTL11* (RM224 and RM144) as described previously (Noenplab et al. 2006). An F_3_ population consisting of 1177 plants for the genetic analysis of *QTL1* was inoculated with a JHN-avirulent isolate BN111. A ratio of 907 resistant versus 270 susceptible progenies was observed, fitting an expected 3:1 ratio (*X* ^2^: 2.665 and *P* value: 0.103) (Additional file 3: Table S2). On the other hand, an F_3_ population comprising of 1346 F_3_ progenies was inoculated with another JHN-avirulent isolate PO6-6 for the genetic analysis of *QTL11*. A ratio of 1024 resistant versus 322 susceptible plants was observed, which fitted an expected 3:1 ratio (*X* ^2^: 0.833, P: 0.361) (Additional file 3: Table S2). These data indicated that the resistance of the F_3_ plants of each population was controlled by a single *R* gene to the isolate BN111 and PO6-6, respectively. Linkage analysis was further conducted to validate the association of *QTL1* and *QTL11* with respective linked markers described previously (Noenplab et al. 2006). Due to no polymorphism resolved between JHN and CO39 at RM319, another polymorphic SSR marker, RM11744 was used instead together with RM212 as flanking markers for the genetic analysis of *QTL1* (Fig. 2a & Additional file 4: Figure S2). All 270 susceptible F_3_ progenies showed CO39-pattern in PCR amplicon size at both flanking markers, suggesting no segregation of *QTL1* from both markers (Fig. 2a). For the linkage analysis of *QTL11*, RM224 and RM144 were used as flanking markers (Fig. 2b & Additional file 4: Figure S2). All 322 susceptible plants showed CO39-pattern in PCR amplicon size at both flanking markers, suggesting that *QTL11* was co-segregated both markers (Fig. 2b). Our data verified that *QTL1* and *QTL11* were delimited into genomic intervals on chromosome 1 and chromosome 11, respectively, as characterized previously (Noenplab et al. 2006).

### QTL1-C and QTL11-C controlled identical resistance spectra as IRBLsh-S and IRBL7-M, respectively

By aligning the positions of flanking SSR markers along respective chromosomes using the Nipponbare reference sequence (MSU Rice Genome Annotation Project, http://rice.plantbiology. msu.edu/), the genomic intervals of *QTL1* and *QTL11* were determined. The genomic interval of *QTL1* corresponded to the region from 33,054,867 bp (RM212) to 33,678,804 bp (RM319) on chromosome 1 in Nipponbare, which is approximately 624 kb in size. Sequence analysis identified 5 predicted NBS-LRR genes in this region, 3 of which corresponded to cloned *R* genes or their alleles, namely *Pi64*, *Pi37*, and *Pish/Pi35* (Table 2). On the other hand, the genomic interval of *QTL11* was conformed to the region from 27,673,251 bp (RM224) to 28,281,693 bp (RM144) on chromosome 11 in Nipponbare, which is approximately 608 kb in size. Nine NBS-LRR genes were predicted in this region, two of which corresponded to the alleles of *Pik-1* and *Pik-2* (Table 2).

**Table 2:** Gene annotation of NBS-LRR gene which located in the genomic intervals of *QTL1* and *QTL11* of JHN using Nipponbare reference sequence

QTL	Chromosome	Chromosomal location of genomic interval (bp)	Annotation of NBS-LRR genes		Allelic genes	References
			NBS-LRR gene model	Chromosomal location (bp)		
*QTL1*	1	33,054,867-33,678,804	LOC_Os01g57270	33,091,703 – 33,096,363	NA	
			LOC_Os01g57280	33,099,464 – 33,103,906	*Pi64*	Ma et al. 2015
			LOC_Os01g57310	33,116,117 – 33,124,371	*Pi37*	Lin et al. 2007
			LOC_Os01g57340	33,141,127 – 33,145,609	*Pish/Pi35*	Takahashi et al. 2010 and Fukuoka et al. 2014
			LOC_Os01g57870	33,463,913 – 33,460,254	NA	
*QTL11*	11	27,673,251-28,281,693	LOC_Os11g45750.1	27,695,070 – 27,683,753	NA	
			XM_015761791.1	27,692,238 – 27,694,418	NA	
			LOC_Os11g45790	27,707,310 – 27,703,761	NA	
			LOC_Os11g45930	27,793,777 – 27,797,821	NA	
			LOC_Os11g45970	27,812,251 – 27,818,431	NA	
			NP_001068480.1	27,821,921 – 27,824,507	NA	
			XM_015760098.1	27,885,376 – 27,886,175	NA	
			LOC_Os11g46200.1	27,978,368 – 27,983,597	*Pik-1* alleles	Ashikawa et al. 2012
			LOC_Os11g46210	27,984,697 – 27,989,134	*Pik-2* alleles	Ashikawa et al. 2012

**Fig. 3:**
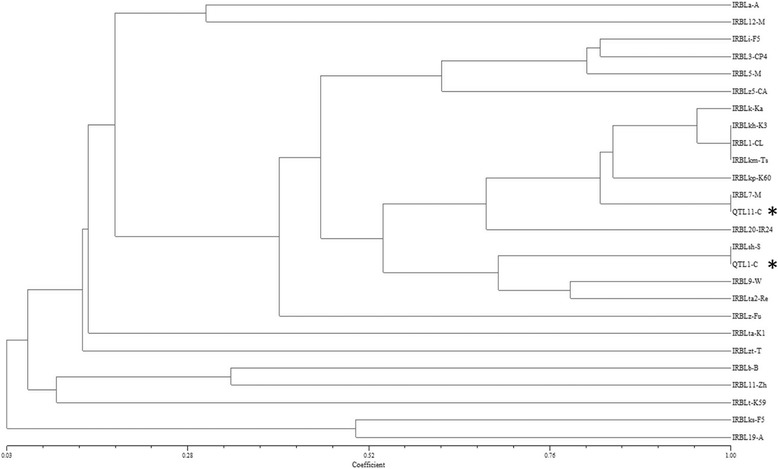
Cluster analysis of disease reaction patterns of QTL1-C, QTL11-C, and IRBLs against 42 representative blast isolates. The lines of QTL1-C and QTL11-C are highlighted in *asterisks*

To validate whether *QTL1* and *QTL11* correspond to any of known *R* genes in respective delimited genomic intervals, we conducted cluster analysis of the resistance spectrum controlled by QTL1-C, QTL11-C, and other IRBLs. Intriguingly, QTL1-C and IRBLsh-S was clustered together while QTL11-C was clustered with IRBL7-M based on their reactions against 42 blast isolates (Fig. 3). This result promoted us to postulate that *QTL1* and *QTL11* in JHN are most likely alleles of *Pish* and *Pi7*, respectively. We thus named them as *Pish-J* and *Pi7-J*.

### *Pish-J* and *Pi7-J* were alleles of known *Pish* and *Pi7*, respectively

To determine the sequence of *Pish-J* and *Pi7-J* in JHN, their genomic regions were cloned and completely sequenced. The sequence of *Pish-J* (GenBank accession number: KY225901) is 4356-bp in length. It contains a predicted 3873-bp coding sequence (CDS), which resulted in a polypeptide product composed of 1290-amino-acids. Sequence comparison revealed that *Pish-J* is identical in nucleotide sequence to *Pish* in Nipponbare (GenBank accession number: AP014957.1). We conclude that *Pish-J* in JHN is an identical allele of *Pish*. Given that *Pik*’s functionality is fulfilled by two adjacent NBS-LRR genes, *Pik-1* and *Pik-2* (Ashikawa et al. 2012), we determined the sequences of their alleles in JHN, designated as *Pi7-J-1* and *Pi7-J-2*, respectively. *Pi7-J-1* is 6489-bp in length (GenBank accession number: KY225902). By referring to sequence annotation of *Pik-1* (GenBank accession number: AB616658), *Pi7-J-1* was predicted to contain three exons interrupted by two introns in the coding region (Additional file 5: Figure S3). It encoded a 1142-amino-acid protein product. *Pi7-J-2* is 3263-bp in length (GenBank accession number: KY225903). It contains two exons and encodes a 1021-amino-acid protein product (Additional file 5: Figure S3).

Sequence comparison revealed that *Pi7-J-1* showed 99% identity in nucleotide sequence with *Pi7-1* (GenBank accession number: HQ660231). It contains two sequence differences along the whole genomic region. One is composed of a single nucleotide “T” deletion at position 1369-bp in the intron and another is a nucleotide transition of G to A at position 5470-bp in the exon 2 of *Pi7-J-1* from *Pi7-1*. The sequence change in the exon belongs to a synonymous substitution and thus does not alter the protein sequence of Pi7-J-1 from Pi7-1. No difference in nucleotide sequences was observed between *Pi7-J-2* and *Pi7-2* (GenBank accession number: HQ660231). Taken together, we concluded that *Pi7-J* represented a new allele of *Pi7* containing two silent mutations in genomic sequence.

The sequence similarity of Pi7-J-1 and Pi7-J-2 with other known Pi7-1 and Pi7-2 alleles was further investigated. It was found that Pi7-J-1 shared 99% identity in amino acid to Pikp-1 and Pikh-1 while it shared 95% identity to Pik-1, Pikm-1, and Pi1-5 (Additional file 6: Table S3). Pi7-J-2 shared 100% identity in amino acid sequence to Pikp-2 and Pikh-2 while it shared 99% sequence identity to Pik-2, Pikm-2, and Pi1-6 (Additional file 6: Table S3)

### The resistance of QTL1-C was controlled by *Pish-J* rather than *Pi64-J* and *Pi37-J*

To exclude the possibility of the contribution of *Pi64* and *Pi37* to the resistance spectrum conferred by *QTL1*, their homologues in JHN, named *Pi64-J* and *Pi37-J*, respectively, were cloned and sequenced. *Pi64-J* was 4406-bp in length covering a predicted 3867-bp CDS while *Pi37-J* was 4474-bp in length covering a predicted 3873-bp CDS. Sequence analysis revealed that Pi64-J and Pi37-J shared 90 and 99% identity in amino acid sequence to Pi64 (Ma et al. 2015) and Pi37 (GenBank accession number: DQ923494.1), respectively, indicating that they were different from known genes. Instead, they were identical in sequence to LOC_Os01g57280 and LOC_Os01g57310, respectively in Nipponbare. Since Nipponbare was used as a reference susceptible variety for both *Pi64* and *Pi37* (Lin et al. 2007; Ma et al. 2015), we thus concluded that *Pish-J* rather than *Pi64-J* and *Pi37-J* was responsible for the resistance of QTL1-C.

## Discussion

The most efficient way to control rice blast disease is the utilization of resistant varieties. It is also low-cost and environment-friendly (Manandhar et al. 1998). The JHN rice variety showed broad-spectrum resistance against Thai and worldwide blast isolates (Sreewongchai 2008). The resistance of JHN was documented in this study by testing it against 132 Philippine blast isolates and found to be effective to most isolates, indicating that it is also quite promising in resistance against rice blast in the Philippines. The dissection of mechanisms underlying the broad-spectrum resistance of JHN against diverse sets of rice blast isolates worldwide is vital for the utilization of JHN-embedded *R* genes in varietal improvement against rice blast via marker aided selection approach. Noenplab et al. (2006)) documented that two QTLs, namely *QTL1* on chromosome 1 and *QTL11* on chromosome 11, were responsible for the broad-spectrum resistance of JHN. In this study, we employed a multifaceted approach to further dissect the molecular mechanisms of both *QTL1* and *QTL11*. The genomic interval spanning *QTL1* and *QTL11* were first delimited within respective regions by flanking markers. Sequence analyses revealed that the *Pish* and *Pik* loci were present in the delimited genomic intervals of *QTL1* and *QTL11*, respectively. Cluster analysis of the resistance reactions of derived *QTL1* and *QTL11* monogenic lines and IRBLs led to the finding of their association with *Pish* and *Pi7*, respectively. Finally, gene cloning and sequence analysis revealed that *QTL1* and *QTL11* encoded alleles of *Pish* and *Pi7*, respectively.

The *Pish* gene was mapped on chromosome 1 by an analysis of QTL due to its moderate resistance and isolated by extensive characterization of retrotransposon-tagged suppressive mutants (Araki et al. 2003; Takahashi et al. 2010). It is located in a gene cluster with a tandem array of four NBS-LRR genes (RGA1 through RGA4) in Nipponbare. It was found that RGA4 rather than RGA1 through RGA3 was responsible for *Pish* (Takahashi et al. 2010). Significant sequence similarity among different homologues suggests that they were likely derived from successive rounds of duplication from a common progenitor (Takahashi et al. 2010). In addition to *Pish*, three other functional *R* genes have been molecularly characterized, namely *Pi64*, *Pi37*, and *Pi35* corresponding to alleles of RGA2, RGA3, and RGA4 in respective resistant rice varieties (Lin et al. 2007; Ma et al. 2015; Fukuoka et al. 2014). Sequence comparison further revealed that multiple functional polymorphisms cumulatively resulted in the conversion from *Pish*-mediated race specific resistance to *Pi35*-mediated non-race specific resistance (Fukuoka et al. 2014). Despite the nature of race-specific resistance, *Pish* was reported to be effective in many Southeast Asian countries including Cambodia, Vietnam, and the Philippines (Fukuta et al. 2014; Nguyen et al. 2015; Selisana et al. 2017).

A total of six *R* genes at the *Pik* locus were characterized at the molecular level including *Pikm* from Tsuyuake (Ashikawa et al. 2008), *Pik* from Kusabue (Zhai et al. 2011), *Pikp* from K60 (Yuan et al. 2011), *Pik* from Kanto51 (Ashikawa et al. 2012), *Pi1* from C101LAC (Hua et al. 2012), and *Pikh* from K3 (Zhai et al. 2014). The functionality of these *Pik* alleles is required by the co-existence of two adjacent NBS-LRR genes at the locus. Different *Pik* alleles activate immunity to rice blast isolates by recognizing different haplotypes of *AvrPik* allele, demonstrating a classical arms race coevolution model (Kanzaki et al. 2012). Biochemical and structural biology assays revealed that the delicate binding between a heavy-metal associated domain (HMA) in Pikp-1 and AvrPik-D initiated the host immune response to rice blast pathogen (Maqbool et al. 2015). In this study, we demonstrated that JHN contained an allele of *Pi7* in IRBL7-M sharing limited sequence differences in nucleotide but not in protein sequence. Resistance spectrum analysis of IRBLs containing different *Pik* alleles against isolates from Bohol clearly indicated that *Pi7*, *Pikp*, and *Pik* controlled resistance to the isolates containing *AvrPik-D* but not other *AvrPik* haplotypes (Selisana et al. 2017). Interestingly, a pathotype test of 2476 rice blast isolates collected from the north, northeast, and south of Thailand in 2007 against IRBLs revealed that IRBLk-ka (*Pik*) and other *Pik*-allele containing IRBLs were resistant to most isolates (Mekwatanakarn et al. 2007). A similar result was obtained in other pathotype tests using 2293 and 1375 Thai rice blast isolates collected in 2011 and 2013, respectively (Mekwatanakarn et al. 2011; Mekwatanakarn et al. 2013). Moreover, *Pi7* was reported to show high frequency of resistance against rice blast isolates in Cambodia (Fukuta et al. 2014) and Vietnam (Nguyen et al. 2015).

In this study, we demonstrated that the resistance spectrum of JHN is attributable to the additive contribution from both *Pi7-J* and *Pish-J*, which underpinned the mechanism of broad-spectrum of resistance to rice blast in JHN. It is indeed that JHN has been widely used as an elite resistant donor in conventional and molecular breeding programs against rice blast disease in Thailand. These breeding programs aim to introgress *QTL1* and *QTL11* of JHN into commercial Thai rice varieties which are susceptible to rice blast. Examples of these varieties are RD6 (Wongsaprom et al. 2010), Khoa Dok Mali 105 (Nalampangnoenplab 2011), IR77955-24-75-284 (Kotchasatit 2013), Ban Tang (Kaewcheenchai et al. 2014), Jao Hawm Phitsanulok51 (Noenplab et al. 2015), and San Par Tong1 (Yajai and Ketsuwan 2015).

## Conclusions

In this study, the *R* genes which control the resistance of *QTL1* and *QTL11* in the JHN rice variety were identified. The *Pish-J* in *QTL1* showed partial resistance while the *Pi7-J* gene in *QTL11* showed complete resistance against Thai and Philippine rice blast isolates. The combination of the two broad spectrum blast resistance genes explains why JHN can still show a high level of resistance for a long period of time in Thailand. The results indicate that *Pish-J* and *Pi7-J* in JHN are broad-spectrum resistance genes which are excellent candidate genes to be included in Thailand and the Philippines's rice breeding pipeline to maintain rice blast resistance.

## Methods

### Plant materials

Jao Hom Nin (JHN), a Thai glutinous rice variety, was used as source of resistance genes for cloning and characterization. CO39, a susceptible rice variety, was used as a recipient variety for genetic linkage analysis. LTH and IRBLs from the Gene Bank of the International Rice Research Institute (IRRI) were used for disease spectrum analysis.

### Generation of monogenic lines of *QTL1* and *QTL11* of JHN in the CO39 genomic background using marker assisted backcrossing (MABC)

The monogenic lines of *QTL1* and *QTL11* of JHN in the CO39 genomic background were generated as illustrated in Additional file 2: Figure S1. In brief, JHN was first crossed with the susceptible rice variety, CO39. The SSR marker RM144 was used to check the real cross of F_1_ plants (Additional file 7: Table S4). Genomic DNA was extracted from frozen rice leaves by CTAB method as described by Doyle and Doyle (1987). To separate *QTL1* and *QTL11*, the resistant F_2_ plants selected by the inoculation of the isolate PO6-6 were screened using SSR markers, RM212 and RM11744 for *QTL1* and RM224 and RM144 for *QTL11* (Additional file 7: Table S4). The homozygous F_2_ plants for individual *QTL1* and *QTL11* were further used for the generation of monogenic lines at the generation of BC_3_F_1_ by MABC. The derived homozygous BC_3_F_3_ plants of *QTL1*, designated as QTL1-C, and of QTL11, designated as QTL11-C were used for spectrum analysis of *QTL1* and *QTL11*, respectively.

### Genetic analysis of QTL1 and QTL11 of JHN

During the generation of monogenic lines of *QTL1* and *QTL11*, the heterozygous F_2_ plants which harbored either *QTL1* or *QTL11* were self-pollinated to produce F_3_ lines for the genetic linkage analysis (Additional file 2: Figure S1). They were inoculated with the differential blast isolates for *Pish* and *Pik*, BN111 and PO6-6, respectively (Telebanco-Yanoria et al. 2008). The DNA of susceptible plants was extracted and used for segregation analysis for *QTL1* with SSR markers of RM212 and RM11744 and for *QTL11* with RM144 and RM224.

### Pathotype analysis

JHN was inoculated with 132 Philippine blast isolates (Additional file 8: Table S5) using 1 × 10^5^ conidia/ml suspension. Pathogen inoculation and disease evaluation were performed as previously described by Zhu et al. (2012). The BC_3_F_3_ homozygous lines of QTL1-C and QTL11-C were inoculated with 42 representative Philippine blast isolates. These 42 representative isolates can be classified in four categories based on the reactions to *Pish* and *Pik* including (I) avirulent to both *Pish* and *Pik*, (II) virulent to both *Pish* and *Pik*, (III) avirulent to *Pish* but virulent to *Pik*, (IV) virulent to *Pish* but avirulent to *Pik*. The disease reactions of QTL1-C and QTL11-C were clustered with those of different IRBLs using NTSYS program version 2.21q.

### Cloning and comparative sequence analysis of known *R* genes in the regions of QTL1 and QTL11 in JHN

The genomic DNA of JHN was used as the DNA template for cloning the homologues of *Pi64/Pi37/Pish* and *Pik* in the regions of *QTL1* and *QTL11* in JHN with the primer pairs listed in Additional file 7: Table S4. PCR amplicons were sequenced (Macrogen Inc, South-Korea) and the online software MAFFT version7 (http://mafft.cbrc.jp/alignment/server/) was used sequence analysis and comparison. Gene and protein sequence similarity was determined via BLASTN and BLASTP, respectively (https://www.ncbi.nlm.nih.gov/). The encoded protein sequences were deduced using the translation program at ExPASy (http://web.expasy.org/translate/).

## Additional files

Additional file 1: Table S1. The resistance reactions of JHN and different IRBLs to 132 Philippines isolates. The 42 representative isolates used for the cluster analysis of *QTL1* and *QTL11* with different target *R* genes in IRBLs were indicated in superscript letter a. IRBL: International Rice Research Institute bred lines; R: resistance; S: susceptible. (DOC 385 kb)

Additional file 2: Figure S1. The diagrammatic flowchart for the generation of mapping population and monogenic lines of QTL1-C and QTL11-C. The heterozygous F2 plants which harbored either QTL1 or QTL11 were selected for self-pollination to produce F3 generation for mapping analysis. (DOC 69 kb)

Additional file 3: Table S2. The genetics of QTL1 and QTL11 in JHN by analyzing F3 population derived from selfing of a single F2 progeny containing either QTL1 or QTL11. (DOC 29 kb)

Additional file 4: Figure S2. Polymorphism analysis of 5 simple sequence repeat (SSR) markers between JHN (J) and CO39 (C). RM224 and RM144 were used as flanking markers for QTL11whereas RM212 and RM11744 was used for QTL1. (DOC 55 kb)

Additional file 5: Figure S3. The gene structure of Pi7-J-1 (a) and Pi7-J-2 (b). The size of each intron and exon was indicated. Black boxes represented exons and lines represented introns. The figure was not drawn in scale. (DOC 43 kb)

Additional file 6: Table S3. Protein sequence similarity of Pi7-J-1 and Pi7-J-2 with other Pi7-1 and Pi7-2 alleles. (DOC 34 kb)

Additional file 7: Table S4. Primers used in this study. (DOC 41 kb)

Additional file 8: Table S5. 132 Philippine rice blast isolates used in the experiment. (DOC 123 kb)
