# Supplementary material for: Dissection of broad-spectrum resistance of the Thai rice variety Jao Hom Nin conferred by two resistance genes against rice blast
Source: Rice (N Y). 2017 May 11;10:18. doi: 10.1186/s12284-017-0159-0 (PMC5425360; doi:10.1186/s12284-017-0159-0)
Supplement: Supplementary file 1 — The resistance reactions of JHN and different IRBLs to 132 Philippines isolates. The 42 representative isolates used for the cluster analysis of QTL1 and QTL11 with different target R genes in IRBLs were indicated in superscript letter a. IRBL: International Rice Research Institute bred lines; R: resistance; S: susceptible. (DOC 385 kb) [file 12284_2017_159_MOESM1_ESM.doc]

| **Table S1** The resistance reactions of JHN and different IRBLs to 132 Philippines isolates. The 42 representative isolates used for the cluster analysis of *QTL1* and *QTL11* with different target *R* genes in IRBLs were indicated in superscript letter a. IRBL: International Rice Research Institute bred lines; R: resistance; S: susceptible | | | | | | | | | | | | | | | | | | | | | | | | | |
| --- | --- | --- | --- | --- | --- | --- | --- | --- | --- | --- | --- | --- | --- | --- | --- | --- | --- | --- | --- | --- | --- | --- | --- | --- | --- |
| Isolate | IRBLs | | | | | | | | | | | | | | | | | | | | | | | | |
|  | IRBLa-A | IRBLi-F5 | IRBLks-F5 | IRBLk-Ka | IRBLkp-K60 | IRBLkh-K3 | IRBLz-Fu | IRBLzt-T | IRBLz5-CA | IRBLta-K1 | IRBLb-B | IRBLt-K59 | IRBLsh-S | IRBL1-CL | IRBL3-CP4 | IRBL5-M | IRBL7-M | IRBL9-W | IRBL12-M | IRBL19-A | IRBLkm-Ts | IRBL20-IR24 | IRBLta2-Re | IRBL11-Zh | JHN |
| 5008-3^a^ | R | R | S | R | R | R | R | S | R | S | S | S | R | R | R | R | R | R | R | S | R | R | R | S | R |
| 5092-3 ^a^ | S | R | S | R | R | R | R | S | R | S | S | S | R | R | R | R | R | R | S | S | R | R | R | S | R |
| 5127-1 | S | R | S | R | R | R | R | S | R | S | S | S | R | R | R | R | R | R | S | S | R | R | R | S | R |
| 5131-2 | S | R | S | R | R | R | R | S | R | S | S | S | R | R | R | R | R | R | S | S | R | R | R | S | R |
| 5167-1 ^a^ | S | S | S | R | R | R | R | R | S | S | S | S | R | R | S | S | S | R | S | S | R | R | S | S | R |
| 6003-3 | R | R | S | R | R | R | R | S | R | S | S | S | R | R | R | R | R | R | R | S | R | R | R | R | R |
| 6006-1 | S | R | S | R | R | R | S | S | R | S | S | S | R | R | R | R | R | R | R | S | R | R | R | R | R |
| 6061-2 | S | R | S | R | R | R | R | S | R | S | S | S | R | R | R | R | R | R | R | S | R | R | R | S | R |
| 6050-3 | S | R | S | R | R | R | R | S | R | S | S | S | R | R | R | R | R | R | R | S | R | R | R | S | R |
| 6161-1 | S | R | S | R | R | R | R | S | R | S | S | S | R | R | R | R | R | R | R | S | R | R | R | R | R |
| 9126-1 | S | R | S | R | R | R | R | S | R | S | S | S | R | R | R | R | R | R | R | S | R | R | R | S | R |
| 9244-3 ^a^ | R | R | S | R | R | R | S | S | R | S | S | S | S | R | R | R | R | R | R | S | R | R | R | S | R |
| 9406-3 ^a^ | S | R | S | R | R | R | R | S | R | S | S | S | R | R | R | R | R | R | R | S | R | R | R | S | R |
| 9475-1 ^a^ | S | S | S | R | R | R | R | S | R | S | S | S | R | R | S | S | R | R | S | S | R | R | R | S | R |
| 9482-1 ^a^ | S | S | S | S | S | R | S | R | R | S | S | S | R | R | S | S | S | R | R | S | R | R | R | S | R |
| 9497-3 ^a^ | R | R | S | R | R | R | R | S | R | S | S | R | S | R | R | R | R | R | R | S | R | R | R | R | R |
| PO6-6 ^a^ | S | S | S | R | R | R | S | S | S | S | S | S | R | R | R | R | R | R | S | S | R | S | S | S | R |
| Ca89 ^a^ | S | S | S | R | R | R | S | S | S | S | S | S | R | R | S | S | R | R | S | S | R | S | S | S | R |
| IK81-3 ^a^ | S | S | S | R | R | R | R | S | S | R | S | S | R | R | S | S | R | R | R | S | R | S | R | S | R |
| IK81-25 ^a^ | S | R | S | S | S | S | R | S | R | R | S | S | R | S | S | R | S | R | R | S | S | S | R | S | R |
| JMB8401 ^a^ | S | R | S | S | S | S | S | S | S | S | S | S | S | S | S | R | S | R | R | S | S | S | R | S | S |
| JMB840610 ^a^ | S | S | S | S | S | S | R | S | R | R | S | S | R | S | S | S | S | R | S | S | S | S | R | S | R |
| M101-1-2-9-1 ^a^ | S | S | S | S | S | S | R | S | S | S | R | S | R | S | S | R | R | R | R | S | S | R | R | R | R |
| M64-1-3-9-1 ^a^ | S | S | S | S | S | S | R | S | S | S | S | S | S | S | S | S | S | R | S | S | S | S | R | S | S |
| PO83-Z1-30 ^a^ | S | S | S | S | S | S | S | S | S | S | S | S | R | S | S | S | R | R | S | S | S | R | R | S | R |
| V86010 ^a^ | S | S | S | R | R | R | S | S | S | S | S | S | R | R | S | S | R | R | S | R | R | R | S | S | R |
| BN111 ^a^ | S | S | S | R | R | R | R | S | R | S | S | S | R | R | R | R | R | R | S | S | R | R | R | S | R |
| BN209 ^a^ | S | S | S | R | R | R | R | S | S | S | S | S | R | R | S | R | R | R | R | S | R | R | R | R | R |
| C9228-37 | S | S | S | R | R | R | R | R | R | S | S | S | R | R | S | S | R | R | S | S | R | S | S | S | R |
| JMB840495 | S | R | S | R | R | R | R | R | R | R | S | S | R | R | R | R | R | R | R | S | R | S | S | S | R |
| V850256 | S | S | S | R | R | R | R | R | R | R | S | S | S | R | S | S | R | R | S | S | R | S | R | S | R |
| MO15-2 ^a^ | S | R | S | R | R | R | S | S | R | S | S | S | R | R | R | R | R | R | S | S | R | R | R | S | R |
| MO15-6 ^a^ | S | R | S | S | R | S | R | S | R | S | S | S | S | S | R | R | S | R | S | S | S | R | R | S | S |
| MO15-20 ^a^ | S | R | S | S | S | S | S | S | R | S | S | R | R | S | R | R | S | R | S | S | S | S | S | S | R |
| MO15-21 ^a^ | S | R | S | R | R | R | S | R | R | R | S | S | R | R | R | R | R | R | S | S | R | R | R | S | R |
| MO15-24 ^a^ | S | S | S | R | R | R | S | S | R | S | S | S | S | R | S | S | R | R | S | S | R | R | R | S | R |
| MO15-32 ^a^ | S | R | S | R | S | R | R | R | R | S | S | S | R | R | R | R | R | R | S | S | R | R | R | S | R |
| MO15-51 ^a^ | S | S | S | R | R | R | S | S | R | S | S | S | R | R | S | S | R | R | S | S | R | R | R | S | R |
| MO15-56 ^a^ | S | R | S | R | R | R | S | S | R | S | S | S | R | R | R | R | R | R | S | S | R | R | R | S | R |
| MO15-64 ^a^ | S | R | S | R | R | R | S | S | S | S | S | S | R | R | R | R | R | R | S | S | R | R | R | S | R |
| MO15-101 | S | R | S | S | R | S | R | R | R | R | S | S | R | S | R | R | S | R | S | R | S | R | S | S | R |
| MO15-102 ^a^ | S | R | S | R | R | R | R | S | R | S | S | S | R | R | R | R | R | R | S | S | R | R | R | S | R |
| MO15-103 | S | R | S | S | S | S | R | S | R | S | R | S | R | S | R | R | S | R | S | S | S | R | R | R | R |
| MO15-104 | S | R | S | S | S | S | R | R | R | R | R | R | R | S | R | R | S | R | R | S | S | S | R | S | R |
| MO15-105 ^a^ | S | R | S | S | S | S | R | S | R | S | S | S | R | S | R | R | S | R | S | S | S | R | R | S | R |
| MO15-106 ^a^ | S | R | S | S | S | S | S | S | R | R | S | R | R | S | R | R | S | R | S | S | S | S | R | S | R |
| MO15-108 | S | R | S | R | R | R | R | S | R | S | S | S | R | R | R | R | R | R | S | S | R | R | R | S | R |
| MO15-110 | S | R | S | S | S | S | R | S | R | S | S | S | R | S | R | R | S | R | S | S | S | R | R | S | R |
| MO15-112 | S | R | R | S | R | S | R | R | S | R | S | S | R | S | R | R | S | R | S | R | S | R | S | S | R |
| MO15-115 | S | R | S | R | R | R | R | S | R | S | R | S | R | R | R | R | R | R | S | S | R | R | R | R | R |
| MO15-116 | S | R | S | S | S | S | R | S | R | S | S | S | R | S | R | R | S | R | S | S | S | R | S | S | R |
| MO15-117 | S | R | S | R | R | R | R | S | R | S | S | S | R | R | R | R | R | R | S | S | R | R | R | S | R |
| MO15-119 | S | R | S | S | S | S | R | S | R | S | R | S | R | S | R | R | S | R | S | S | S | R | R | R | R |
| MO15-120 | S | R | S | S | S | S | R | S | R | R | S | S | R | S | R | R | S | R | S | S | S | R | R | S | R |
| MO15-121 | S | R | R | S | R | S | R | R | R | R | S | S | R | S | R | R | S | R | S | R | S | R | S | S | R |
| MO15-124 | S | R | S | S | S | S | R | S | R | S | S | S | R | S | R | R | S | R | S | S | S | R | S | S | R |
| MO15-125 | S | R | S | S | S | S | R | R | R | S | S | S | R | S | R | R | S | R | S | S | S | S | S | S | R |
| MO15-126 | S | R | S | S | S | S | R | S | R | S | S | S | R | S | R | R | S | R | S | S | S | R | R | S | R |
| MO15-127 | S | R | S | R | R | R | S | S | R | S | S | S | R | R | R | R | R | R | S | S | R | R | R | S | R |
| MO15-129 | S | R | S | R | R | R | S | S | R | S | S | S | R | R | R | R | R | R | S | S | R | R | R | S | R |
| MO15-130 | S | R | R | S | R | S | R | R | R | R | S | S | R | S | R | R | S | R | S | R | S | R | S | S | R |
| MO15-131 | S | R | R | S | R | S | R | R | R | R | S | S | R | S | R | R | S | R | S | R | S | S | S | S | R |
| MO15-132 | S | R | S | S | R | S | R | R | S | R | S | S | R | S | R | R | S | R | S | R | S | R | S | S | R |
| MO15-133 | S | R | S | R | R | R | S | S | R | S | S | S | R | R | R | R | R | R | S | S | R | R | R | S | R |
| MO15-134 | S | R | S | S | S | S | R | S | R | S | S | S | R | S | R | R | S | R | S | S | S | R | R | S | R |
| MO15-136 | S | R | S | S | S | S | R | S | R | S | S | S | R | S | R | R | S | R | S | S | S | R | R | S | R |
| MO15-137 | S | R | S | S | S | S | S | S | R | S | S | S | R | S | R | R | S | R | S | S | S | R | R | S | R |
| MO15-138 ^a^ | S | R | S | S | S | S | S | R | R | S | S | S | S | S | R | R | S | R | S | S | S | S | S | S | S |
| MO15-144 ^a^ | S | R | S | S | S | S | R | S | R | S | S | S | R | S | R | R | S | R | S | S | S | R | R | S | R |
| MO15-145 | S | R | S | S | S | S | S | S | R | S | S | S | R | S | R | R | S | R | S | S | S | R | R | S | R |
| MO15-146 | S | R | R | R | R | R | S | S | R | S | S | S | R | R | R | R | R | R | S | S | R | R | R | S | R |
| MO15-148 ^a^ | S | R | S | S | S | S | S | R | R | S | S | S | S | S | R | R | S | R | S | S | S | S | S | S | S |
| MO15-150 | S | R | S | R | R | R | R | S | R | S | S | S | R | R | R | R | R | R | S | S | R | R | R | S | R |
| MO15-151 | S | R | S | R | R | R | R | S | R | S | S | S | R | R | R | R | R | R | S | S | R | R | R | S | R |
| MO15-152 | S | R | R | S | R | S | R | R | S | R | S | S | R | S | R | R | S | R | S | R | S | S | S | S | R |
| MO15-153 | S | R | S | R | R | R | R | S | R | S | S | S | R | R | R | R | R | R | S | S | R | R | R | S | R |
| MO15-154 | S | R | S | R | R | R | R | S | R | S | S | S | R | R | R | R | R | R | S | S | R | R | R | S | R |
| MO15-155 | S | R | S | R | R | R | R | S | R | S | S | S | R | R | R | R | R | R | S | S | R | R | R | S | R |
| MO15-158 | S | R | S | R | R | R | R | S | R | S | S | S | R | R | R | R | R | R | S | S | R | R | R | S | R |
| MO15-159 | R | R | S | R | R | R | R | S | R | R | R | R | R | R | R | R | R | R | S | S | R | R | R | R | R |
| MO15-160 | S | R | R | S | S | S | R | R | R | R | S | S | R | S | R | R | S | R | R | R | S | R | R | S | R |
| MO15-161 | S | R | S | S | S | S | R | S | R | R | R | S | R | S | R | R | S | R | R | S | S | S | R | S | R |
| MO15-162 | S | R | S | S | S | S | S | S | R | S | S | S | R | S | R | R | S | R | S | S | S | R | R | S | R |
| MO15-164 | S | R | R | S | R | S | R | R | S | R | S | S | R | S | R | R | S | R | S | R | S | R | R | S | R |
| MO15-168 | S | R | R | S | R | S | R | R | R | R | S | S | S | S | R | R | S | R | S | R | S | R | S | S | S |
| MO15-170 | S | R | S | S | R | S | S | R | S | R | S | S | R | S | R | R | S | R | S | R | S | R | S | S | R |
| MO15-171 | S | R | S | S | S | S | S | S | R | R | S | S | R | S | R | R | S | R | S | S | S | R | S | S | R |
| MO15-172 | S | R | S | S | R | S | R | R | R | R | S | R | R | S | R | R | S | R | S | R | S | R | S | S | R |
| MO15-174 | S | R | S | R | R | R | R | R | R | R | R | R | R | R | R | R | R | R | S | S | R | R | R | R | R |
| MO15-175 | S | R | R | R | R | R | R | S | R | R | R | S | R | R | R | R | R | R | S | S | R | R | R | S | R |
| MO15-176 | S | R | R | S | R | S | R | R | R | R | S | S | R | S | R | R | S | R | S | R | S | R | S | S | R |
| MO15-181 | S | R | S | R | R | R | R | S | R | S | S | S | R | R | R | R | R | R | S | S | R | R | R | S | R |
| MO15-182 | S | R | S | S | S | S | R | S | R | S | S | S | R | S | R | R | S | R | S | S | S | R | R | S | R |
| MO15-184 | S | R | S | S | R | S | R | R | R | R | S | R | R | S | R | R | S | R | S | R | S | R | S | S | R |
| MO15-185 | S | R | S | R | R | R | S | S | R | S | S | S | R | R | R | R | R | R | S | S | R | R | S | S | R |
| MO15-188 | S | R | S | S | R | S | R | R | R | R | S | S | R | S | R | R | S | R | S | R | S | R | S | S | R |
| MO15-189 | S | R | S | S | R | S | R | R | R | R | S | S | R | S | S | S | S | R | S | R | S | R | S | S | R |
| MO15-190 | S | R | S | S | R | S | R | R | R | R | S | S | R | S | R | R | S | R | S | R | S | R | S | S | R |
| MO15-191 | S | R | R | S | S | S | R | R | R | R | S | S | R | S | R | R | S | R | S | R | S | R | S | S | R |
| MO15-192 | S | R | S | R | R | R | R | S | R | S | S | S | R | R | R | R | R | R | S | S | R | R | R | S | R |
| MO15-193 | S | R | R | S | R | S | R | R | R | R | S | S | R | S | R | R | S | R | S | R | S | R | S | S | R |
| MO15-194 | S | R | S | S | R | S | R | R | R | R | S | R | R | S | R | R | S | R | S | R | S | R | S | S | R |
| MO15-195 | S | R | S | S | S | S | R | R | R | S | S | S | R | S | R | R | S | R | S | S | S | S | S | S | R |
| MO15-196 | R | R | R | R | R | R | R | S | R | S | R | R | R | R | R | R | R | R | S | S | R | R | R | R | R |
| MO15-197 | S | R | S | S | R | S | S | R | R | R | S | S | R | S | R | R | S | R | S | R | S | S | S | R | R |
| MO15-199 | S | R | S | R | R | R | R | S | R | S | S | S | R | R | R | R | R | R | S | S | R | S | R | S | R |
| MO15-200 ^a^ | S | R | R | S | R | S | R | R | R | R | S | S | R | S | R | R | S | R | S | R | S | R | S | S | R |
| Pi9-G7-1V-1 ^a^ | S | S | S | S | S | S | R | S | R | R | S | S | R | S | S | R | S | R | S | S | S | S | R | S | R |
| Pi9-G7-1I-1 ^a^ | S | S | S | S | S | S | R | S | S | R | S | S | R | S | S | S | S | R | S | S | S | S | R | S | R |
| Pi9-G7-3D-1 | S | S | S | S | S | S | R | S | R | R | S | S | R | S | S | R | S | R | S | S | S | S | R | S | R |
| Pi9-G7-2I-1 | S | S | S | S | S | S | R | S | R | R | S | S | R | S | S | S | S | R | S | S | S | S | R | S | R |
| Pi9-G7-2A-1 | S | S | S | S | S | S | R | S | R | R | S | S | R | S | S | S | S | R | S | S | S | S | R | S | R |
| Pi9-G7-1H-1 ^a^ | S | S | S | S | S | S | S | S | R | R | S | S | R | S | S | S | S | R | S | S | S | S | R | S | R |
| Pi9-G7-1W-1 ^a^ | S | S | S | S | S | S | R | S | R | R | S | S | S | S | S | S | S | R | S | S | S | S | S | S | S |
| Pi9-G7-1L-1 | S | S | S | S | S | S | R | S | S | R | S | S | R | S | S | R | S | R | S | S | S | S | R | S | R |
| Pi9-G7-1F-1 | S | S | S | S | S | S | R | S | R | R | S | S | R | S | S | S | S | R | S | S | S | S | R | S | R |
| Pi9-G7-1N-1 | S | S | S | S | S | S | R | S | S | R | S | S | R | S | S | S | S | R | S | S | S | S | R | S | R |
| Pi9-G9-C-2 | S | S | S | S | S | S | R | S | S | R | S | S | R | S | S | R | S | R | S | S | S | S | R | S | R |
| Pi9-G7-1D-1 | S | S | S | S | S | S | R | S | S | R | S | S | R | S | S | S | S | R | S | S | S | S | R | S | R |
| Pi9-G7-2E-1 | S | S | S | S | S | S | R | S | R | R | S | S | R | S | S | S | S | R | S | S | S | S | R | S | R |
| Pi9-G7-1P-1 | S | S | S | S | S | S | R | S | S | R | S | S | S | S | S | S | S | R | S | S | S | S | R | S | S |
| Pi9-G7-1K-1 | S | S | S | S | S | S | S | S | R | S | S | S | R | S | S | S | S | R | S | S | S | S | S | S | R |
| Pi9-G7-3A-1 | S | S | S | S | S | S | R | S | S | R | S | S | R | S | S | R | S | R | S | S | S | S | R | S | R |
| Pi9-G7-2G-1 | S | S | S | S | S | S | R | S | S | R | S | S | R | S | S | S | S | R | S | S | S | S | R | S | R |
| Pi9-G7-1J-1 | S | S | S | S | S | S | R | S | R | R | S | S | R | S | S | R | S | R | S | S | S | S | R | S | R |
| Pi9-G7-1A-1 | S | S | S | S | S | S | R | S | R | R | S | S | S | S | S | S | S | R | S | S | S | S | R | S | S |
| Pi9-G7-2K-1 | S | S | S | S | S | S | R | S | R | R | S | S | R | S | S | S | S | R | S | S | S | S | R | S | R |
| Pi9-G7-1Q-1 | S | S | S | S | S | S | R | S | R | R | S | S | R | S | S | S | S | R | S | S | S | S | R | S | R |
| Pi9-G9-1E-1 | S | S | S | S | S | S | R | S | R | R | S | S | R | S | S | S | S | R | S | S | S | S | R | S | R |
| Pi9-G7-1B-1 ^a^ | S | S | S | S | S | S | R | S | R | R | S | S | S | S | S | S | S | R | S | S | S | S | R | S | S |
| Pi9-G7-2F-1 | S | S | S | S | S | S | R | S | R | R | S | S | S | S | S | S | S | R | S | S | S | S | R | S | S |
| Pi9-G7-1E-1 ^a^ | S | S | S | S | S | S | R | S | S | S | S | S | S | S | S | S | S | R | S | S | S | S | R | S | S |
| Resistance frequency (%) | 4.54 | 68.18 | 11.36 | 40.15 | 55.3 | 40.9 | 76.51 | 26.51 | 81.06 | 44.69 | 7.57 | 7.57 | 87.87 | 40.9 | 67.42 | 75 | 40.9 | 100 | 15.15 | 17.42 | 40.9 | 64.39 | 71.96 | 9.84 | 90.9 |
|  |  |  |  |  |  |  |  |  |  |  |  |  |  |  |  |  |  |  |  |  |  |  |  |  |  |
