# Supplementary material for: Dissection of broad-spectrum resistance of the Thai rice variety Jao Hom Nin conferred by two resistance genes against rice blast
Source: Rice (N Y). 2017 May 11;10:18. doi: 10.1186/s12284-017-0159-0 (PMC5425360; doi:10.1186/s12284-017-0159-0)
Supplement: Supplementary file 2 — The diagrammatic flowchart for the generation of mapping population and monogenic lines of QTL1-C and QTL11-C. The heterozygous F2 plants which harbored either QTL1 or QTL11 were selected for self-pollination to produce F3 generation for mapping analysis. (DOC 69 kb) [file 12284_2017_159_MOESM2_ESM.doc]

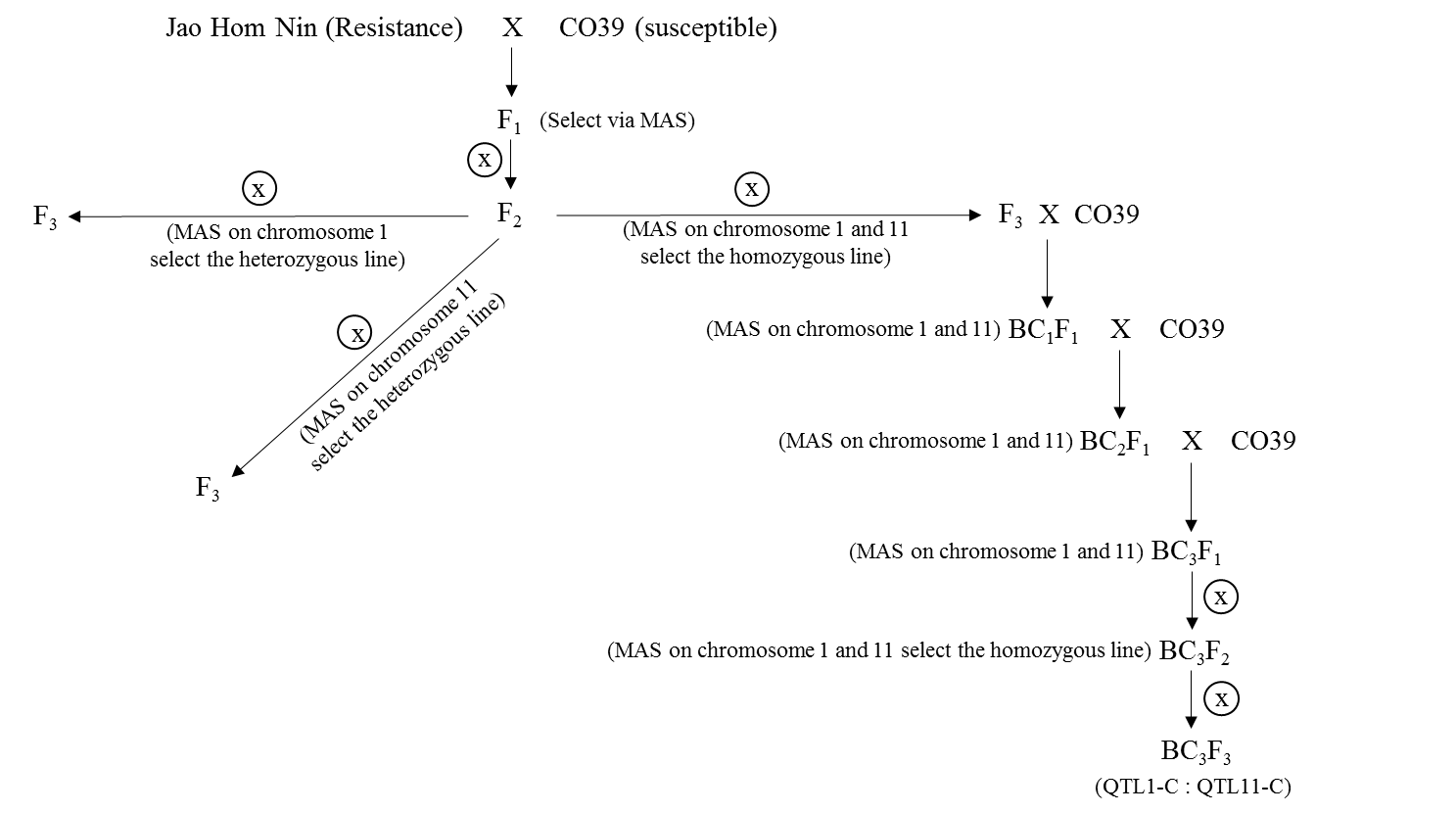


**Figure S1** The diagrammatic flowchart for the generation of mapping population and monogenic lines of QTL1-C and QTL11-C. The heterozygous F_2_ plants which harbored either *QTL1* or *QTL11* were selected for self-pollination to produce F_3_ generation for mapping analysis. The homozygous F_2_ lines of either *QTL1* or *QTL11* were used for the generation of BC_3_F_2_ by marker aided backcrossing (MABC). The BC_3_F_3_ homozygous plants were used for the disease resistance assessment. CO39 was used as the recipient line. Four SSR markers were used for the marker aided selection (MAS) for *QTL1* (RM212 and RM11744) and *QTL11* (RM224 and RM144) as indicated in Additional file 7: Table S4.
