# Supplementary material for: Dissection of broad-spectrum resistance of the Thai rice variety Jao Hom Nin conferred by two resistance genes against rice blast
Source: Rice (N Y). 2017 May 11;10:18. doi: 10.1186/s12284-017-0159-0 (PMC5425360; doi:10.1186/s12284-017-0159-0)
Supplement: Supplementary file 3 — The genetics of QTL1 and QTL11 in JHN by analyzing F3 population derived from selfing of a single F2 progeny containing either QTL1 or QTL11. (DOC 29 kb) [file 12284_2017_159_MOESM3_ESM.doc]

***Table S2*** *The genetics of QTL1 and QTL11 in JHN by analyzing F_3_ population derived from selfing of a single F_2_ progeny containing either QTL1 or QTL11*

| QTLs | Blast isolates used for phenotyping | Number of F_3_ progenies | | Chi square analysis based on an expected 3(R):1(S) ratio | |
| --- | --- | --- | --- | --- | --- |
|  |  | Resistant (R) | Susceptible (S) | X^2^ | P value |
| *QTL1* | BN111 | 907 | 270 | 2.665 | 0.103 |
| *QTL11* | PO6-6 | 1,024 | 322 | 0.833 | 0.361 |
