# Supplementary material for: Dissection of broad-spectrum resistance of the Thai rice variety Jao Hom Nin conferred by two resistance genes against rice blast
Source: Rice (N Y). 2017 May 11;10:18. doi: 10.1186/s12284-017-0159-0 (PMC5425360; doi:10.1186/s12284-017-0159-0)
Supplement: Supplementary file 4 — Polymorphism analysis of 5 simple sequence repeat (SSR) markers between JHN (J) and CO39 (C). RM224 and RM144 were used as flanking markers for QTL11whereas RM212 and RM11744 was used for QTL1. (DOC 55 kb) [file 12284_2017_159_MOESM4_ESM.doc]

**
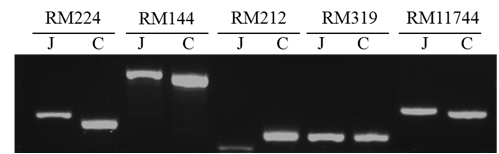
**

**Figure S2** Polymorphism analysis of 5 simple sequence repeat (SSR) markers between JHN (J) and CO39 (C). RM224 and RM144 were used as flanking markers for *QTL11*whereas RM212 and RM11744 was used for *QTL1*. Another SSR marker RM319 did not show polymorphism in size between JHN and CO39. The PCR products were resolved in 5% of agarose gel.
