# Supplementary material for: Dissection of broad-spectrum resistance of the Thai rice variety Jao Hom Nin conferred by two resistance genes against rice blast
Source: Rice (N Y). 2017 May 11;10:18. doi: 10.1186/s12284-017-0159-0 (PMC5425360; doi:10.1186/s12284-017-0159-0)
Supplement: Supplementary file 5 — The gene structure of Pi7-J-1 (a) and Pi7-J-2 (b). The size of each intron and exon was indicated. Black boxes represented exons and lines represented introns. The figure was not drawn in scale. (DOC 43 kb) [file 12284_2017_159_MOESM5_ESM.doc]

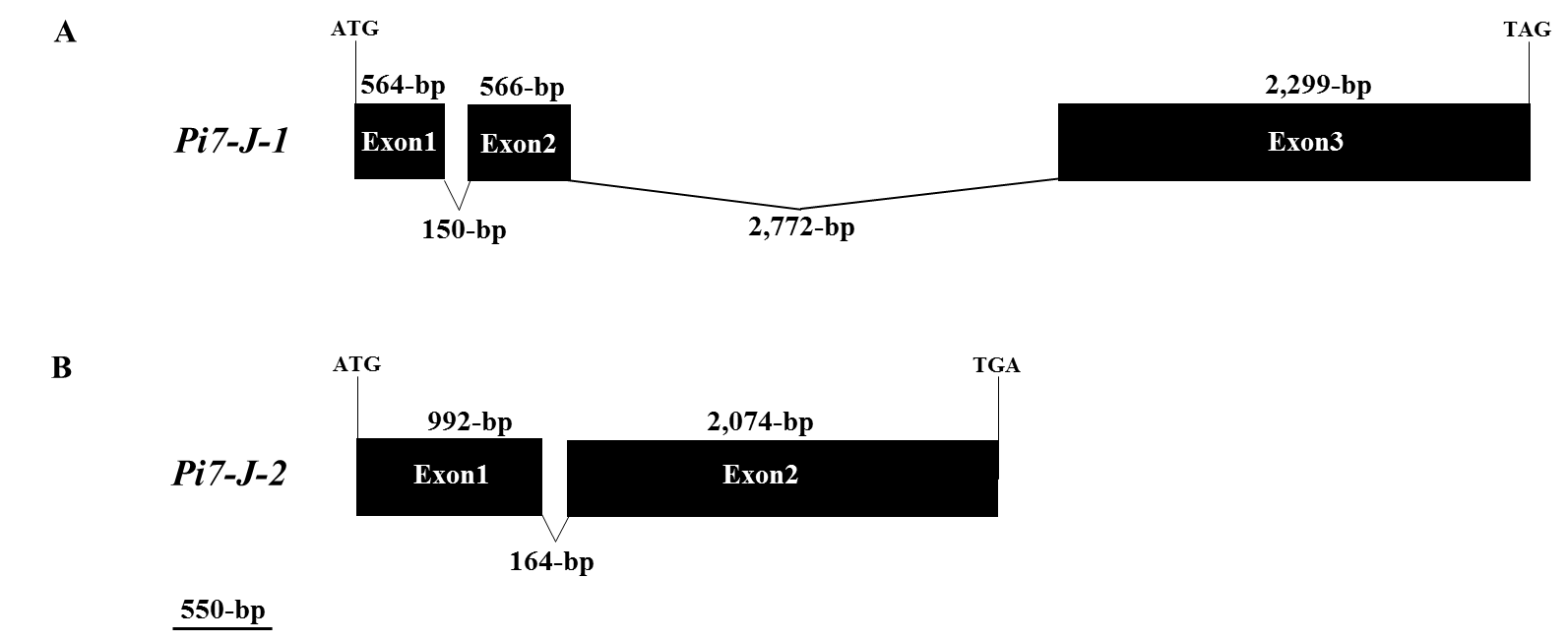


**Figure S3** The gene structure of *Pi7-J-1* **(a)** and *Pi7-J-2* **(b)**. The size of each intron and exon was indicated. Black boxes represented exons and lines represented introns. The figure was not drawn in scale.
