# Supplementary material for: Dissection of broad-spectrum resistance of the Thai rice variety Jao Hom Nin conferred by two resistance genes against rice blast
Source: Rice (N Y). 2017 May 11;10:18. doi: 10.1186/s12284-017-0159-0 (PMC5425360; doi:10.1186/s12284-017-0159-0)
Supplement: Supplementary file 6 — Protein sequence similarity of Pi7-J-1 and Pi7-J-2 with other Pi7-1 and Pi7-2 alleles. (DOC 34 kb) [file 12284_2017_159_MOESM6_ESM.doc]

Table S3 Protein sequence similarity of Pi7-J-1 and Pi7-J-2 with other Pi7-1 and Pi7-2 alleles

| Pi7-J | Pi7-J alleles | GenBank accession number | Similarity (%) |
| --- | --- | --- | --- |
| Pi7-J-1 | Pi7-1 | AET36551.1 | 100 |
|  | Pikp-1 | ADV58352.1 | 99 |
|  | Pikh-1 | AET36549.1 | 99 |
|  | Pik-1 | ADZ48537.1 | 95 |
|  | Pikm-1 | BAG72135.1 | 95 |
|  | Pi1-5 | AEB00617.1 | 95 |
| Pi7-J-2 | Pi7-2 | AET36552.1 | 100 |
|  | Pikp-2 | ADV58351.1 | 100 |
|  | Pikh-2 | AET36550.1 | 100 |
|  | Pik-2 | ADZ48538.1 | 99 |
|  | Pikm-2 | BAG72136.1 | 99 |
|  | Pi1-6 | AEB00618.1 | 99 |
