# Supplementary material for: Dissection of broad-spectrum resistance of the Thai rice variety Jao Hom Nin conferred by two resistance genes against rice blast
Source: Rice (N Y). 2017 May 11;10:18. doi: 10.1186/s12284-017-0159-0 (PMC5425360; doi:10.1186/s12284-017-0159-0)
Supplement: Supplementary file 7 — Primers used in this study. (DOC 41 kb) [file 12284_2017_159_MOESM7_ESM.doc]

| **Table S4** Primers used in this study | | | |
| --- | --- | --- | --- |
| Primers | Sequence (5′-3′) | Tm (^o^C) | Purpose |
| RM212F | CCACTTTCAGCTACTACCAG | 58 | Check the inheritance of *Pish* locus |
| RM212R | CACCCATTTGTCTCTCATTATG | 58 | Check the inheritance of *Pish* locus |
| RM319F | ATCAAGGTACCTAGACCACCAC | 58 | Check the inheritance of *Pish* locus |
| RM319R | TCCTGGTGCAGCTATGTCTG | 58 | Check the inheritance of *Pish* locus |
| RM11744F | CCACCCGTATAGGACCAGTCG | 58 | Check the inheritance of *Pish* locus |
| RM11744R | TAGAGTCTCCAGGCAGTCTCACC | 58 | Check the inheritance of *Pish* locus |
| RM224F | ATCGATCGATCTTCACGAGG | 58 | Check the inheritance of *Pik* locus |
| RM224R | TGCTATAAAAGGCATTCGGG | 58 | Check the inheritance of *Pik* locus |
| RM144F | TGCCCTGGCGCAAATTTGATCC | 58 | Check the inheritance of *Pik* locus |
| RM144R | GCTAGAGGAGATCAGATGGTAGTGCATG | 58 | Check the inheritance of *Pik* locus |
| Pish-5UTR-F6 | CACGCTTTCTTGTCTCTGCT | 58 | Clone the full length of *Pish-J* gene |
| Pish-3UTL-R6 | CTATATGAGCATGAGGCGCT | 58 | Clone the full length of *Pish-J* gene |
| Pi37-5UTR-F5 | CCATCACCATCTGTTCTTAT | 58 | Clone the full length of *Pi37-J* gene |
| Pi37-3UTL-R3 | GGGTTCCACCATATATATACC | 58 | Clone the full length of *Pi37-J* gene |
| Pi64-F1 | GTCCGGCTAGAATATCTTAG | 58 | Clone the full length of *Pi64-J* gene |
| Pi64-R1 | ATATGTGCCAATCTTGGGGT | 58 | Clone the full length of *Pi64-J* gene |
| 3RGA4F | AATTAAGGCGCGCCAGAGGTGCAAATTCCTAACG | 60 | Clone the full length of *Pi7-J-1* gene |
| RGA4R | TTTAAATTAATTAAAGAGTATTACATGAAGGAGAG | 60 | Clone the full length of *Pi7-J-1* gene |
| 3RGA5F | AATTAAGGCGCGCCTCCTCTCGCAATGGGTTAGAG | 58 | Clone the full length of *Pi7-J-2* gene |
| RGA5R | TTTAAATTAATTAAGGGAAGCCTCAGATGATTGG | 58 | Clone the full length of *Pi7-J-2* gene |
