# Supplementary material for: Dissection of broad-spectrum resistance of the Thai rice variety Jao Hom Nin conferred by two resistance genes against rice blast
Source: Rice (N Y). 2017 May 11;10:18. doi: 10.1186/s12284-017-0159-0 (PMC5425360; doi:10.1186/s12284-017-0159-0)
Supplement: Supplementary file 8 — 132 Philippine rice blast isolates used in the experiment. (DOC 123 kb) [file 12284_2017_159_MOESM8_ESM.doc]

**Table S5** 132 Philippine rice blast isolates used in the experiment.

| Isolates | Host varieties | Locations for the collection | Year collected |
| --- | --- | --- | --- |
| 5008-3 | IR11A501 | Ubay, Bohol | 2014 |
| 5092-3 | PR40096-9-1-1 | Ubay, Bohol | 2014 |
| 5127-1 | PR 38086-B-31-B-B-B | Ubay, Bohol | 2014 |
| 5131-2 | PR 40094-35-1 | Ubay, Bohol | 2014 |
| 5167-1 | IR11A583 | Ubay, Bohol | 2014 |
| 6003-3 | IR09N542 | Ubay, Bohol | 2014 |
| 6006-1 | PR40094-35-1 | Ubay, Bohol | 2014 |
| 6061-2 | PR40858-NSIC Rc 9-M4R-437 | Ubay, Bohol | 2014 |
| 6050-3 | PR34350-2-POKKALI-AC-24-M5R-8 (DRS 87) | Ubay, Bohol | 2014 |
| 6161-1 | PR380-86-B-31-B-B-B | Ubay, Bohol | 2014 |
| 9126-1 | IR101465-10-7 | Ubay, Bohol | 2014 |
| 9244-3 | IR13A268 | Ubay, Bohol | 2014 |
| 9406-3 | IR95624-B-71-1-B-B | Ubay, Bohol | 2014 |
| 9475-1 | NSIC Rc 158 | Ubay, Bohol | 2014 |
| 9482-1 | IR12N269 | Ubay, Bohol | 2014 |
| 9497-3 | NSIC Rc 158 | Ubay, Bohol | 2014 |
| PO6-6 | Tetep | IRRI | 1980 |
| Ca89 | IR 50 | Caliraya, Laguna | 1990 |
| IK81-3 | Milyang 49 | IRRI | 1981 |
| IK81-25 | Milyang 49 | unknown | 1981 |
| JMB8401 | IR29725-3-1-3 | Caliraya, Laguna | 1985 |
| JMB840610 | UPLRi-5 | Cuenca, Batangas | 1984 |
| M101-1-2-9-1 | C101A51 | IRBN | 1995 |
| M64-1-3-9-1 | IR 64 | IRBN | 1995 |
| PO83-Z1-30 | unknown | unknown | unknown |
| V86010 | unknown | Camarines Sur | 1986 |
| BN111 | IR47686-18-7B | IRBN | 1990 |
| BN209 | unknown | unknown | unknown |
| C9228-37 | unknown | unknown | unknown |
| JMB840495 | unknown | unknown | unknown |
| V850256 | UPLRi-5 | Blast Nursery | 1985 |
| MO15-2 | IRBLb-IT13[CO] | Ubay, Bohol | 2014 |
| MO15-6 | IRBLkm-Ts[CO] | Ubay, Bohol | 2014 |
| MO15-20 | IRBLkp-K60[CO] | Ubay, Bohol | 2014 |
| MO15-21 | 117622 | Ubay, Bohol | 2014 |
| MO15-24 | 121514 | Ubay, Bohol | 2014 |
| MO15-32 | IR93336:49-B-10-14-B-B-B-44 | Ubay, Bohol | 2014 |
| MO15-51 | IR12A287 | Ubay, Bohol | 2014 |
| MO15-56 | IR96856-97-3-1 | Ubay, Bohol | 2014 |
| MO15-64 | IR11A583 | Ubay, Bohol | 2014 |
| MO15-101 | IRRI156 | Ubay, Bohol | 2015 |
| MO15-102 | IR66 | Ubay, Bohol | 2015 |
| MO15-103 | IRBLkh-K3[CO] | Ubay, Bohol | 2015 |
| MO15-104 | IRBL1-CL | Ubay, Bohol | 2015 |
| MO15-105 | IRBLk-Ku[CO] | Ubay, Bohol | 2015 |
| MO15-106 | IRBLkh-K3 | Ubay, Bohol | 2015 |
| MO15-108 | IRBLta-Ya[CO] | Ubay, Bohol | 2015 |
| MO15-110 | IRBLkm-Ts[CO] | Ubay, Bohol | 2015 |
| MO15-112 | IRRI123 | Ubay, Bohol | 2015 |
| MO15-115 | IRBLks-F5 | Ubay, Bohol | 2015 |
| MO15-116 | IRBLZT-IR56[CO] | Ubay, Bohol | 2015 |
| MO15-117 | IRRI165 | Ubay, Bohol | 2015 |
| MO15-119 | IRRI161 | Ubay, Bohol | 2015 |
| MO15-120 | IRBL1-CL[CO] | Ubay, Bohol | 2015 |
| MO15-121 | IRBLta2-Re[CO] | Ubay, Bohol | 2015 |
| MO15-124 | IRBL7-M[CO] | Ubay, Bohol | 2015 |
| MO15-125 | IRBLks-CO [CO] | Ubay, Bohol | 2015 |
| MO15-126 | IRBLkp-K60 | Ubay, Bohol | 2015 |
| MO15-127 | IR29 | Ubay, Bohol | 2015 |
| MO15-129 | CO39 | Ubay, Bohol | 2015 |
| MO15-130 | IRRI136 | Ubay, Bohol | 2015 |
| MO15-131 | IRRI118 | Ubay, Bohol | 2015 |
| MO15-132 | IR64-SUB1 | Ubay, Bohol | 2015 |
| MO15-133 | IR52 | Ubay, Bohol | 2015 |
| MO15-134 | IRBL7-M | Ubay, Bohol | 2015 |
| MO15-136 | IRRI133 | Ubay, Bohol | 2015 |
| MO15-137 | IRRI138 | Ubay, Bohol | 2015 |
| MO15-138 | IR8 | Ubay, Bohol | 2015 |
| MO15-144 | IRBLkp-K60[CO] | Ubay, Bohol | 2015 |
| MO15-145 | IRBLb-IT13[CO] | Ubay, Bohol | 2015 |
| MO15-146 | IRRI134 | Ubay, Bohol | 2015 |
| MO15-148 | IRBLzt-T | Ubay, Bohol | 2015 |
| MO15-150 | CO39 | Ubay, Bohol | 2015 |
| MO15-151 | IRBLa-A | Ubay, Bohol | 2015 |
| MO15-152 | IRBLta2-Pi | Ubay, Bohol | 2015 |
| MO15-153 | IR38 | Ubay, Bohol | 2015 |
| MO15-154 | CO39 | Ubay, Bohol | 2015 |
| MO15-155 | IR65 | Ubay, Bohol | 2015 |
| MO15-158 | IRBL5-M[CO] | Ubay, Bohol | 2015 |
| MO15-159 | IR28 | Ubay, Bohol | 2015 |
| MO15-160 | IRBL12-M | Ubay, Bohol | 2015 |
| MO15-161 | IRBLta2-IR64[CO] | Ubay, Bohol | 2015 |
| MO15-162 | IRRI160 | Ubay, Bohol | 2015 |
| MO15-164 | IR36 | Ubay, Bohol | 2015 |
| MO15-168 | IRRI149 | Ubay, Bohol | 2015 |
| MO15-170 | IRRI118 | Ubay, Bohol | 2015 |
| MO15-171 | IR28 | Ubay, Bohol | 2015 |
| MO15-172 | IRBLkm-Ts | Ubay, Bohol | 2015 |
| MO15-174 | IR65 | Ubay, Bohol | 2015 |
| MO15-175 | IRBLz5-CA[CO] | Ubay, Bohol | 2015 |
| MO15-176 | IRBLt-K59 | Ubay, Bohol | 2015 |
| MO15-181 | IR72 | Ubay, Bohol | 2015 |
| MO15-182 | IRBLkm-Ts-CO] | Ubay, Bohol | 2015 |
| MO15-184 | IR60 | Ubay, Bohol | 2015 |
| MO15-185 | IR93336:49-B-10-14-B-B-B-2 | Ubay, Bohol | 2015 |
| MO15-188 | IR12A177 | Ubay, Bohol | 2015 |
| MO15-189 | CIHERANG-SUB1 | Ubay, Bohol | 2015 |
| MO15-190 | IR12N193 | Ubay, Bohol | 2015 |
| MO15-191 | IR12A196 | Ubay, Bohol | 2015 |
| MO15-192 | IR12A152 | Ubay, Bohol | 2015 |
| MO15-193 | IR64-SUB1 | Ubay, Bohol | 2015 |
| MO15-194 | IR12A299 | Ubay, Bohol | 2015 |
| MO15-195 | IR12N198 | Ubay, Bohol | 2015 |
| MO15-196 | IR93336:49-B-10-14-B-B-B-20 | Ubay, Bohol | 2015 |
| MO15-197 | IR93339:29-B-7-7-B-B-B-37 | Ubay, Bohol | 2015 |
| MO15-199 | IR13N159 | Ubay, Bohol | 2015 |
| MO15-200 | IR12A108 | Ubay, Bohol | 2015 |
| Pi9-G7-1V-1 | WHD-1S-75-1-127 | IRRI | 2014 |
| Pi9-G7-1I-1 | WHD-1S-75-1-127 | IRRI | 2014 |
| Pi9-G7-3D-1 | WHD-1S-75-1-127 | IRRI | 2014 |
| Pi9-G7-2I-1 | WHD-1S-75-1-127 | IRRI | 2014 |
| Pi9-G7-2A-1 | WHD-1S-75-1-127 | IRRI | 2014 |
| Pi9-G7-1H-1 | WHD-1S-75-1-127 | IRRI | 2014 |
| Pi9-G7-1W-1 | WHD-1S-75-1-127 | IRRI | 2014 |
| Pi9-G7-1L-1 | WHD-1S-75-1-127 | IRRI | 2014 |
| Pi9-G7-1F-1 | WHD-1S-75-1-127 | IRRI | 2014 |
| Pi9-G7-1N-1 | WHD-1S-75-1-127 | IRRI | 2014 |
| Pi9-G9-C-2 | WHD-1S-75-1-127 | IRRI | 2014 |
| Pi9-G7-1D-1 | WHD-1S-75-1-127 | IRRI | 2014 |
| Pi9-G7-2E-1 | WHD-1S-75-1-127 | IRRI | 2014 |
| Pi9-G7-1P-1 | WHD-1S-75-1-127 | IRRI | 2014 |
| Pi9-G7-1K-1 | WHD-1S-75-1-127 | IRRI | 2014 |
| Pi9-G7-3A-1 | WHD-1S-75-1-127 | IRRI | 2014 |
| Pi9-G7-2G-1 | WHD-1S-75-1-127 | IRRI | 2014 |
| Pi9-G7-1J-1 | WHD-1S-75-1-127 | IRRI | 2014 |
| Pi9-G7-1A-1 | WHD-1S-75-1-127 | IRRI | 2014 |
| Pi9-G7-2K-1 | WHD-1S-75-1-127 | IRRI | 2014 |
| Pi9-G7-1Q-1 | WHD-1S-75-1-127 | IRRI | 2014 |
| Pi9-G9-1E-1 | WHD-1S-75-1-127 | IRRI | 2014 |
| Pi9-G7-1B-1 | WHD-1S-75-1-127 | IRRI | 2014 |
| Pi9-G7-2F-1 | WHD-1S-75-1-127 | IRRI | 2014 |
| Pi9-G7-1E-1 | WHD-1S-75-1-127 | IRRI | 2014 |
